# Risk Without Reward: Differing Patterns of Chemotherapy Use Do Not Improve Outcomes in Stage II Early-Onset Colon Cancer

**DOI:** 10.1200/OP.24.00159

**Published:** 2024-07-24

**Authors:** Jacob B. Leary, Junxiao Hu, Alexis Leal, S. Lindsey Davis, Sunnie Kim, Robert Lentz, Tyler Friedrich, Whitney Herter, Wells A. Messersmith, Christopher H. Lieu

**Affiliations:** ^1^ Department of Medicine, University of Washington, Seattle, WA; ^2^ Biostatistics Shared Resource, University of Colorado Cancer Center, Aurora, CO; ^3^ Division of Medical Oncology, University of Colorado Anschutz Medical Campus, Aurora, CO; ^4^ Department of Surgery, University of Colorado Anschutz Medical Campus, Aurora, CO

## Abstract

**PURPOSE:**

Rising rates of early-onset colon cancer (EOCC) present challenges in deciding how to optimally treat patients. Although standard of care for stage II CC is surgical resection, adding chemotherapy for high-risk disease, evidence suggests treatment selection may differ by age. We investigated whether adjuvant chemotherapy (AC) administration rates differ between patients with early- and later-onset stage II CC.

**METHODS:**

Data originated from the nationwide Flatiron Health electronic health record (EHR)–derived deidentified database spanning January 1, 2003, to August 1, 2021. Adults with stage II CC were grouped as age 18-49 years (EOCC) and those age 50 years or older (later-onset colon cancer [LOCC]). Demographics, Eastern Cooperative Oncology Group score, tumor stage and site, and chemotherapy were included. Primary outcomes included rates of AC administration by age and ethnicity; secondary outcomes included overall survival (OS) and time to metastatic disease (TTMD). Univariate and multivariable logistic regression models evaluated relationships between chemotherapy administration, age, and ethnicity, adjusting for significant covariates.

**RESULTS:**

One thousand sixty-five patients were included. Median age of patients with EOCC was 45.0 years versus 69.0 years for patients with LOCC. Adjusted multivariate analysis showed patients with EOCC received AC significantly more often than patients with LOCC. Non-Hispanic patients received AC at significantly lower rates than Hispanic patients in both cohorts. Subanalysis of stage IIA patients showed that patients with EOCC were more likely to receive AC than patients with LOCC. No significant differences in OS or TTMD were observed by age regardless of AC administration in stage II overall; however, patients with stage IIA EOCC receiving AC had significantly longer TTMD than those not receiving AC.

**CONCLUSION:**

AC was given preferentially in stage II EOCC, even in stage IIA, despite deviation from guidelines. This may expose low-risk patients to unnecessary toxicities and suggests bias toward treating younger patients more aggressively, despite unclear evidence for better outcomes.

## INTRODUCTION

Colorectal cancer (CRC) is the third most common cancer diagnosed in the United States and ranks as the second leading cause of cancer-related death, with approximately 52,550 associated deaths projected in 2023.^1^ Despite long being considered a disease of later adulthood, early-onset CRC (EOCRC) occurring in those younger than 50 years is becoming increasingly common. Incidence rates in the United States have been steadily rising since the mid-1990s, with 5.9 cases per 100,000 persons in 2000 versus 8.4 cases per 100,000 persons in 2017.^2^ Such changes in the presentation of CRC pose new challenges for clinicians when determining the best treatment course to maximize outcomes.

**CONTEXT**

**Key Objective**
This work aimed to investigate disparities in postresection adjuvant chemotherapy (AC) treatment patterns between patients initially diagnosed with early-onset versus later-onset stage II colon cancer (CC).
**Knowledge Generated**
This study demonstrated bias in treatment practices for stage II CC, revealing a higher rate of AC use in young versus older patients, even in stage IIA disease where guidelines do not routinely support AC. A higher rate of AC treatment in these patients was not associated with better outcomes, including no overall survival benefit and a negligible difference in time to metastatic disease.
**Relevance**
Given the increased risk of potential adverse events and unnecessary toxicities associated with chemotherapy use, providers should work to avoid preferential chemotherapy use in patients with early-onset CC as there is no clear benefit achieved from this practice. This could help to lower risks for patients with early-onset CC and improve quality of life.

Standard of care for stage II CRC differs on the basis of primary site of disease. Stage II rectal cancer guidelines recommend neoadjuvant or adjuvant chemoradiation in all cases except for highly differentiated, noninvasive tumors.^3^ Conversely, guideline-based treatment for stage II CC is largely surgical resection with curative intent, except in high-risk cases of stage IIB/IIC disease where adjuvant chemotherapy may be indicated.^4^ Despite these evidence-based recommendations, adjuvant chemotherapy is offered to approximately 20% of patients with stage II CC regardless of whether high-risk features are present.^5^ Given an overall favorable prognosis after surgical resection with 5-year disease-free survival of 68%-83%^6^ and relatively low risk of recurrence, a substantial proportion of these patients will experience chemotherapy-related toxicities, financial burden, and unnecessary disruptions to daily life without experiencing benefits from treatment.

Evidence has also emerged to suggest that treatment selection may differ by patient age such that younger patients with stage II CC may be treated more aggressively than older patients, even when controlling for clinicopathologic differences representing higher-risk disease.^7^ In this study by Hagerty et al, rates of treatment decreased in a stepwise manner across age groups, such that the youngest patients were treated with AC most frequently while very low rates of AC were provided to those older than 75 years. Kneuertz and et al^8^ similarly demonstrated that younger patients receive AC more frequently than older patients across all stages, with no survival benefit observed in those with stage II disease. These findings raise concern for age-related bias in treatment patterns, with deviation from guideline-based treatment failing to improve outcomes in young patients with stage II CC while disproportionately raising their risk of adverse effects from chemotherapy exposure.

Our work focused on investigating disparities in postresection AC treatment patterns between younger and older patients with initially diagnosed stage II CC using a large metastatic colorectal cancer database, expanding upon previous studies by comparing rates between age groups in stage II disease overall and also specifically within stage IIA disease where AC is not recommended. We hypothesized that younger patients would receive adjuvant chemotherapy more often than their older counterparts in both stage II disease overall and in stage IIA disease. Secondarily, we sought to evaluate differences in patient outcomes on the basis of whether patients received adjuvant chemotherapy. We hypothesized that overall survival (OS) and time to metastatic disease (TTMD) would not differ between groups, regardless of chemotherapy administration.

## METHODS

### Patient Selection

Patients included in our study were adults age 18 years or older diagnosed with stage II CC with eventual progression to metastatic disease. All patients were derived from the nationwide Flatiron Health electronic health record (EHR)–derived deidentified database, a longitudinal database comprising deidentified patient-level structured and unstructured data curated via technology-enabled abstraction. The majority of patients in the database originate from community oncology settings; relative community/academic proportions may vary depending on study cohort. The data are deidentified and subject to obligations to prevent reidentification and protect patient confidentiality. During the study period spanning 1/1/2003 to 8/1/2021, the deidentified data originated from approximately 280 US cancer clinics (approximately 800 sites of care). A cohort of patients were initially diagnosed with stage II CC. We then divided patients into two groups: early-onset colon cancer (EOCC), defined as those who were diagnosed before age 50 years, and later-onset colon cancer (LOCC), which included those diagnosed at age 50 years or later. Age used to categorize patients was calculated using birth year and year of initial diagnosis.

Patients were excluded if they had >90 days between initial diagnosis and first electronic health record structured data entry (n = 6,816). We excluded patients with rectal cancer (n = 706) as chemotherapy administration guidelines differ between colon and rectal cancers. We also excluded patients with missing values for variables of interest (n = 362).

### Outcomes of Interest

Demographic characteristics of interest in our study included median age, sex, race, Hispanic or Latino versus non-Hispanic or Latino ethnicity, Eastern Cooperative Oncology Group (ECOG) score for functional status, stage of disease (II, IIA, IIB, and IIC), and site of disease (colon or colorectal-not otherwise specified [NOS]). ECOG score was taken from the ECOG record associated with either the initial diagnosis date or a visit immediately after the initial diagnosis date, whichever was available. Microsatellite status was also evaluated in a subset of patients who had these data available, classifying patients as either microsatellite stable (MSS) or microsatellite instability—high (MSI-H). MSI-H status has been shown to correlate with a more favorable prognosis in CC.^9,10^ The rationale for examining this was to determine any contribution of differing rates of MSI-H status to the likelihood of patients in either group receiving chemotherapy, which may have implications for our findings.

The primary clinical outcome for this study was rate of chemotherapy administration in stage II disease, expressed as percentage of patients in each group who received adjuvant chemotherapy. Patients were considered to have received chemotherapy if they had received at least one dose of any of the following between the initial diagnosis date and the date of diagnosis of metastatic disease: oxaliplatin, leucovorin, levoleucovorin, fluorouracil, capecitabine, irinotecan, or bevacizumab. Secondary outcomes of interest included OS and TTMD, both measured in months from time of initial diagnosis of CC. For OS, the patients who had not experienced the event before the last day of follow-up were censored. Per inclusion criteria, all patients have progressed to metastatic disease, thus no patients were censored for TTMD.

### Statistical Analysis

Univariate and multivariable logistic regression models were used to evaluate relationships between chemotherapy administration, age groups, and ethnicity while adjusting for significant covariates. An additional subanalysis was conducted to examine rates of chemotherapy administration in stage IIA patients. Sensitivity analysis using 1:1 propensity score matching (PSM) with nearest neighbor matching without replacement was conducted to balance age groups for important covariates of interest including sex, ethnicity, ECOG score, and site of disease for all stage II patients and stage IIA patients only. Logistic regression models (univariate) were then used to compare chemotherapy administration between the age groups in the PSM data set. Log-rank tests were conducted to compare OS and TTMD among groups. The Kaplan-Meier method was used to calculate survival curves and 95% CIs.

## RESULTS

### Patient Demographics by Group

A total of 1,065 patients with stage II CC were included. Of these, 105 were patients with EOCC and 960 were patients with LOCC. Patient demographic information and cancer-related characteristics by group are detailed in Table 1.

**TABLE 1.** Patient Demographics and Cancer-Related Characteristics

Variable	EOCC (n = 105)	LOCC (n = 960)
Age, years, median (IQR)	45.0 (41.0-47.0)	69.0 (61.0-76.0)
Male, No. (%)	49 (46.7)	517 (53.9)
Female, No. (%)	56 (53.3)	443 (46.1)
Ethnicity, No. (%)		
Hispanic or Latino	8 (7.6)	69 (7.2)
Non-Hispanic	97 (92.4)	891 (92.8)
Race, No. (%)		
Asian	5 (4.8)	22 (2.3)
Black/African American	14 (13.3)	115 (12.0)
Hispanic or Latino	0 (0)	5 (0.5)
Other	23 (21.9)	115 (12.0)
White	63 (60.0)	703 (73.2)
ECOG, No. (%)		
0	71 (67.6)	497 (51.8)
1	31 (29.5)	337 (35.1)
>1	3 (2.9)	126 (13.1)
Stage, No. (%)		
II*	7 (6.7)	74 (7.7)
IIA	59 (56.2)	614 (64.0)
IIB	30 (28.6)	182 (19.0)
IIC	9 (8.6)	90 (9.4)
Site, No. (%)		
Colon	103 (98.1)	953 (99.3)
Colorectal, NOS	2 (1.9)	7 (0.7)
Microsatellite status, No. (%)^a^		
MSS	42 (87.5)	295 (90.2)
MSI-H	6 (12.5)	32 (9.8)

Abbreviations: ECOG, Eastern Cooperative Oncology Group; EOCC, early-onset colon cancer; LOCC, later-onset colon cancer; MSI-H, microsatellite instability-high; MSS, microsatellite stable; NOS, not otherwise specified.

^a^ Microsatellite status was available for 375 patients.

### Primary Outcome—Chemotherapy Administration Rates Between Groups

Table 2 displays comparisons of chemotherapy administration rates between groups. Overall, the majority of patients in stage II and stage IIA CC cohorts did not receive AC. Significantly higher rates of chemotherapy administration were observed for patients with EOCC compared with those with LOCC (37.1% *v* 21.9%, *P* < .001) in the overall stage II cohort. This observation held true in the subanalysis of patients with stage IIA disease (*P* = .015). Patients matched via PSM reflected a similar pattern in both stage II disease (*P* = .002) and stage IIA disease (*P* = .019); for details on PSM matched data, see Appendix Table A1 (online only). Notably, in this cohort, patients who identified as non-Hispanic/Latino were less likely to receive chemotherapy than those who identified as Hispanic/Latino in both stage II disease (*P* = .018) and stage IIA disease (*P* < .001). For full details, see Table 2.

**TABLE 2.** Adjuvant Chemotherapy Administration Rates

Analysis Group	Received AC, No. (%)	Did Not Receive AC, No. (%)	Adjusted OR^a^ (95% CI)	*P*
Stage II EOCC	39 (37.1)	66 (62.9)	2.02 (1.31 to 3.09)	**<.001**
Stage II LOCC	210 (21.9)	750 (78.1)	—	—
PSM stage II EOCC	39 (37.1)	66 (62.9)	2.67 (1.43 to 5.13)	**.002**
PSM stage II LOCC	19 (18.1)	86 (81.9)	—	—
Stage IIA EOCC	15 (25.4)	44 (74.6)	2.20 (1.13 to 4.08)	**.015**
Stage IIA LOCC	85 (13.8)	529 (86.2)	—	—
PSM stage IIA EOCC	15 (25.4)	44 (74.6)	3.68 (1.31 to 12.04)	**.019**
PSM stage IIA LOCC	5 (8.5)	54 (91.5)	—	—

	Hispanic	Non-Hispanic	Adjusted OR^b^ (95% CI)	*P*
Stage II Hispanic/Latino	27 (35.1)	50 (64.9)	0.55 (0.34 to 0.91)	**.018**
Stage II Non-Hispanic/Latino	222 (22.5)	766 (77.5)	—	—
Stage IIA Hispanic/Latino	17 (30.9)	38 (69.1)	0.34 (0.18 to 0.64)	**<.001**
Stage IIA Non-Hispanic/Latino	83 (13.4)	535 (86.6)	—	—

NOTE. Bolded text indicates statistical significance.

Abbreviations: AC, adjuvant chemotherapy; ECOG, Eastern Cooperative Oncology Group; EOCC, early-onset colon cancer; LOCC, later-onset colon cancer; OR, odds ratio; PSM, propensity score matching.

^a^ Adjusted for ethnicity, sex, site, and ECOG score.

^b^ Adjusted for age, sex, site, and ECOG score.

### Microsatellite Status Subanalysis

Microsatellite status was available for 375 patients in our cohort, of whom 228 had stage IIA disease. The EOCC group contained six patients (12.5%) with MSI-H disease and 42 patients (87.5%) with MSS disease, while the LOCC group contained 32 patients (9.8%) with MSI-H disease and 295 patients (90.2%) with MSS disease. Multivariate analysis adjusting for sex, ethnicity, site, and ECOG score revealed that rates of chemotherapy administration were not significantly different for MSS versus MSI-H patients (MSS = 26.4% *v* MSI-H = 26.3%, *P* = .99). Microsatellite status also did not emerge as a significant covariate when comparing rates of chemotherapy administration, TTMD, or OS between groups.

### Secondary Outcomes—TTMD and OS by Group

**FIG 1.**
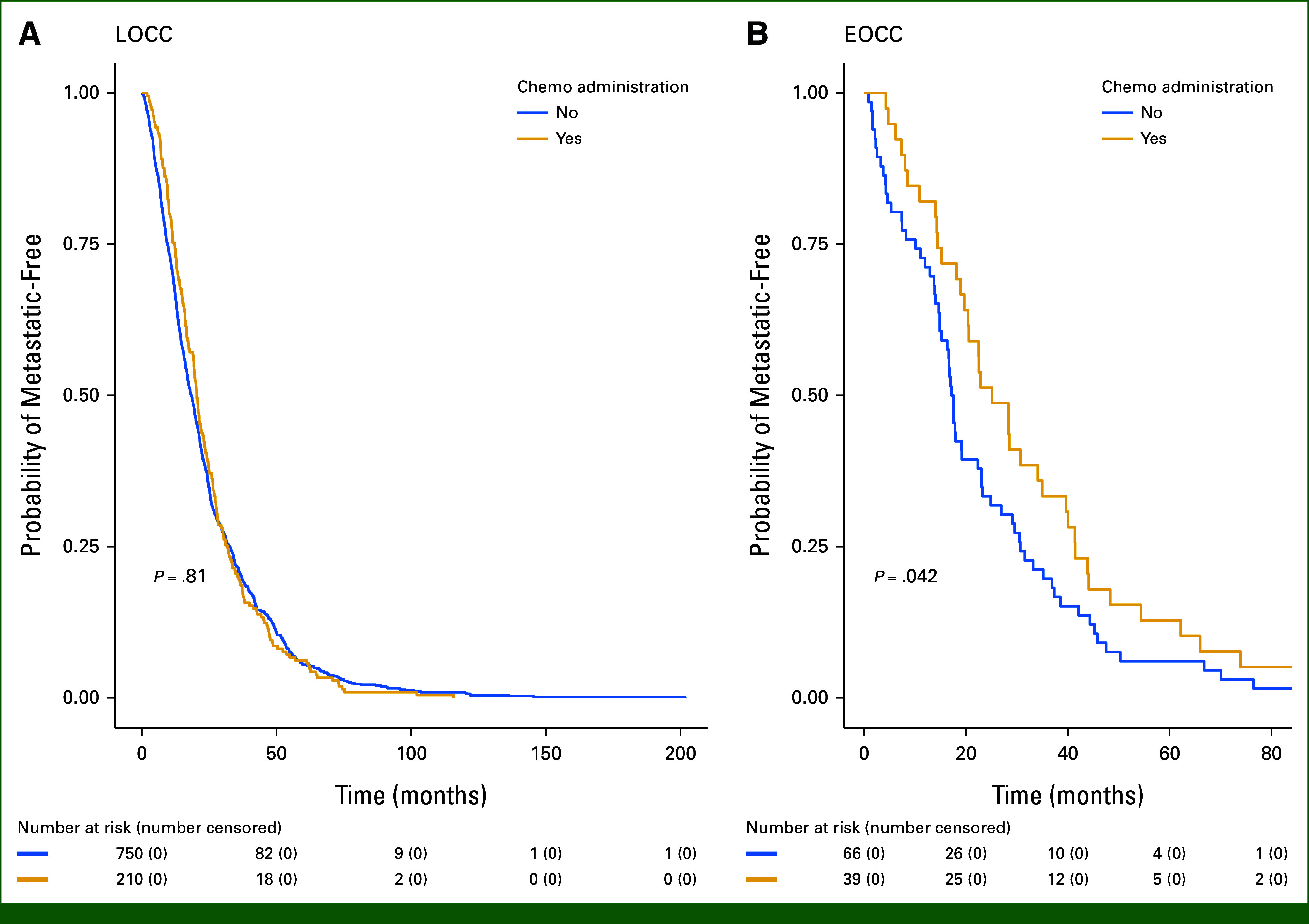
Kaplan-Meier curves for TTMD comparing chemotherapy administration by group: (A) LOCC group and (B) EOCC group. Figure 1 illustrates TTMD for each group, comparing those receiving chemotherapy with those who did not. For the EOCC group, longer TTMD was seen with AC administration; for the LOCC group, AC administration did not significantly affect TTMD. AC, adjuvant chemotherapy; EOCC, early-onset colon cancer; LOCC, later-onset colon cancer; TTMD, time to metastatic disease.

Median TTMD by age group was 19.1 months (95% CI, 17.1 to 24.8) for EOCC and 19.2 months for LOCC (95% CI, 17.5 to 20.3; *P* = .490). Median probability of being metastatic-free at 1 year for all patients was 70.3% (95% CI, 67.6 to 73.1). No significant differences were observed in TTMD regardless of whether or not patients received chemotherapy in the LOCC group (*P* = .81). However, TTMD was significantly longer in the EOCC group in those who received chemotherapy versus those who did not (25.1 months [95% CI, 20.4 to 39.7]) versus (17.3 months [95% CI, 14.8 to 23.1], *P* = .042). Median follow-up time was 19.2 months. Figure 1 displays Kaplan-Meier curves for TTMD.

**FIG 2.**
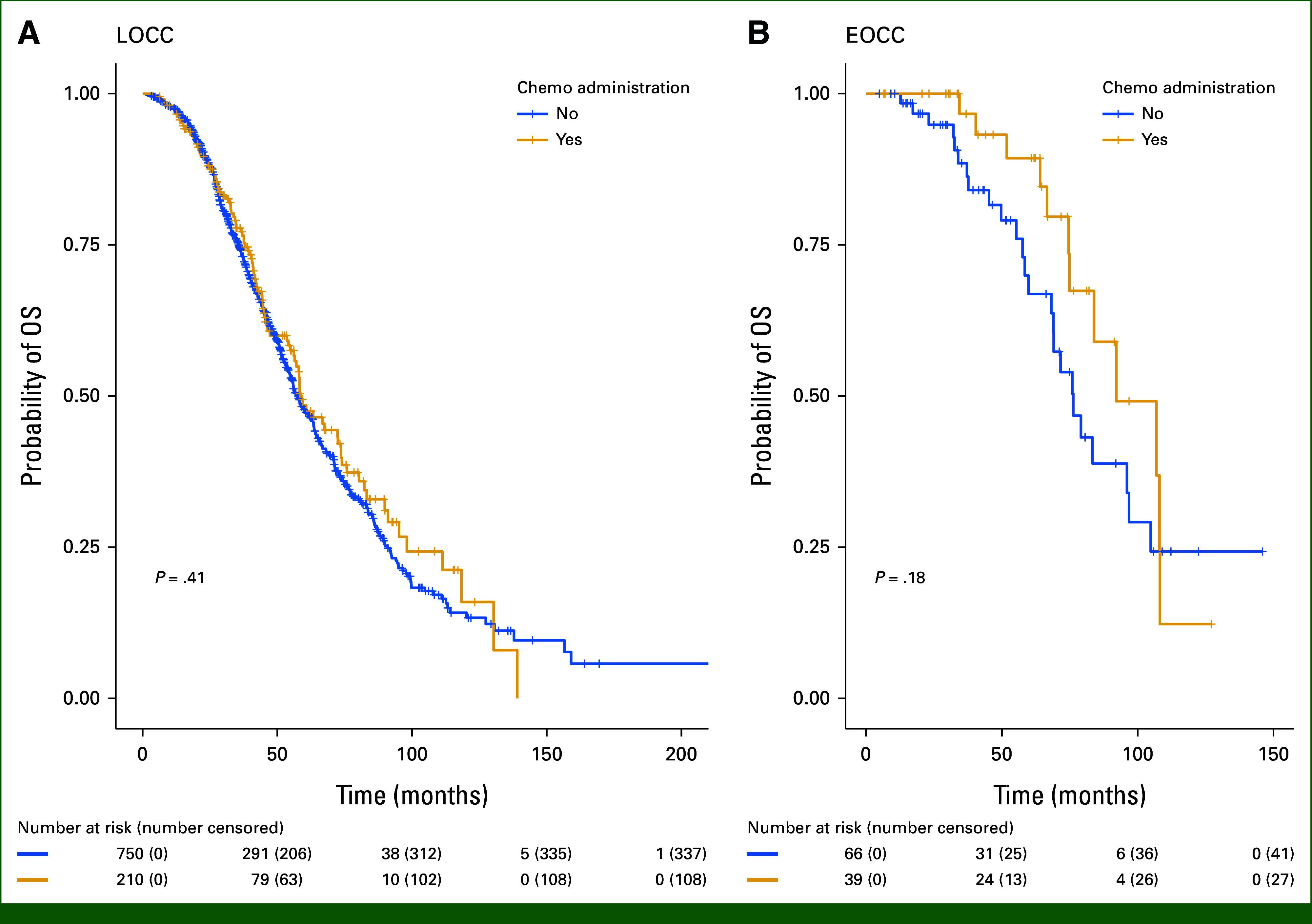
Kaplan-Meier curves for OS comparing chemotherapy administration by group: (A) LOCC group and (B) EOCC group. Figure 2 shows OS for each group on the basis of whether patients did or did not receive chemotherapy. No significant differences were observed in OS for each group regardless of chemotherapy administration. EOCC, early-onset colon cancer; LOCC, later-onset colon cancer; OS, overall survival.

At the time of analysis, 552 patients had died (EOCC = 37, LOCC = 515) and 513 patients were alive. For those who died, median survival for EOCC was significantly longer at 83.4 months (95% CI, 74.9 to 106.9) compared with LOCC at 58.2 months (95% CI, 55.8 to 63.3; *P* < .0001). When comparing OS for each group, 76.2% (95% CI, 66.8 to 87.0) of patients with EOCC were still alive at 5 years after diagnosis versus 47.8% (95% CI, 44.2 to 51.8; *P* < .00001) of patients with LOCC. No significant differences were observed among either patients with EOCC (*P* = .18) or patients with LOCC (*P* = .41) regardless of whether or not patients received chemotherapy. Median follow-up time was 41.6 months. Figure 2 includes Kaplan-Meier curves for OS by group.

## DISCUSSION

Our work sought to replicate the findings of several recent studies suggesting differential rates of AC administration in stage II CC between patients with EOCC and patients with LOCC. In a cohort of stage II patients who eventually developed metastatic disease, we showed that the majority of patients are not treated with AC, in accordance with guidelines. However, in the smaller but substantial proportion of patients who do undergo chemotherapy, those with EOCC received AC more frequently than patients with LOCC. We expanded on previous works by demonstrating that a bias toward more aggressive treatment holds true even in stage IIA disease where AC is not recommended. In our cohort, we also noted higher rates of AC given to patients of Hispanic ethnicity compared with non-Hispanic individuals. Despite higher rates of AC administration in younger patients, we showed no difference in OS regardless of chemotherapy treatment status. We demonstrated a small but significant difference in TTMD in the EOCC group between those who did and did not receive AC; in the LOCC group, no differences in TTMD were observed.

Increasing evidence suggests that younger patients with stage II CC are treated more aggressively after resection than older patients,^7,8,11^ even in node-negative disease, and this appears to occur in a graded fashion such that the youngest patients receive AC most often while the oldest patients are offered AC least often.^7^ Our findings support this phenomenon of discordant treatment practices between younger and older patients with CC. AC is recommended on a case-by-case basis in high-risk stage IIB and IIC disease,^4^ so there are situations in which AC is reasonable on the basis of an individual patient's tumor profile. Although limitations in our data set precluded us from being able to specifically identify high-risk tumor characteristics among these patients, the differential rates in AC administration cannot be attributed solely to higher-risk tumors in younger patients as the treatment bias persisted even in stage IIA disease where AC is not indicated per guidelines. Moreover, rates of MSI in our patients were within the established frequency of 8%-20% observed in the CC population at large^9,12^ and were not significantly different between EOCC and LOCC cohorts. This argues against the possibility that AC was provided differentially between groups on the basis of the known association between MSI-H tumors and more favorable outcomes, as well as lower likelihood of response to certain chemotherapies in these tumors.

Skewed treatment practices may in part be explained by mixed findings from earlier studies that showed potential improvements in outcomes including longer recurrence-free survival, disease-free survival, and OS with AC in stage II patients.^13-19^ Some data have also suggested lack of benefit from AC in patients age 70 years and older with stage II CC.^16,20^ However, the studies suggesting support for AC in stage II disease either failed to show statistically and clinically significant benefit or were limited by study design, such as combining stage II and stage III patients into one cohort, combining patients with colon and rectal cancers, or were influenced by a stage migration effect, calling into question the validity of their findings. We also acknowledge the unique concerns that must be considered when deciding on the appropriateness of chemotherapy for older patients, including a higher burden of other medical comorbidities, reduced social support, and potentially impaired functional status that may reduce the ability to deliver chemotherapy safely. These geriatric factors likely contribute to why older patients are treated less frequently with AC across disease stages. However, across multiple phase III trials, the outcome benefits achieved with AC were not mitigated by age, and significant adverse event rates were similar between younger and older patients.^17,20-22^ If physicians are going to offer AC to patients against guideline recommendations, this practice must be equitable and AC should be offered at a similar frequency to older patients when appropriate. It is our strong recommendation, however, that practitioners adhere to stage II guidelines not to treat routinely with AC after resection given the increasing evidence, including the findings of this work, against any benefit achieved through such practices.

Although an expected difference was observed in OS between EOCC and LOCC cohorts that can likely be attributed to age-related factors, we found no difference in OS between patients who received AC and those who did not in both the EOCC and LOCC groups. All patients in our sample eventually developed metastatic disease, which likely played a substantial role in shaping OS outcomes. However, it is important to highlight that provision of chemotherapy did not alter the eventual survival statistics, as this suggests that more aggressive intervention during the stage II disease period may not increase a patient's chance of being alive at 5-year follow-up should they go on to develop metastatic CC.

We also observed no difference in TTMD in older adults with or without AC; conversely, we saw a significantly longer TTMD in young adults treated with AC compared with their untreated counterparts. This argues against our hypothesis, and if true would warrant further investigation as more time without metastatic disease may translate to a longer time period over which disease can be treated with curative intent, greater ability to return to work,^23^ and fewer hospitalizations,^24^ which lowers financial costs and allows for more time engaged in activities meaningful to the patient. However, we suspect this observation may be more likely a result of the relatively small sample of individuals receiving AC in the EOCC group as this raises the risk that outliers may skew the distribution. This possibility is supported by the wider CI for TTMD data observed in this subsample when compared with patients with EOCC who did not receive AC. Further investigation in a larger sample is needed to confirm this observation.

Nonetheless, our findings largely align with those of Kneuertz et al,^8^ showing no measurable benefit to survival in treating stage II CC with AC. In this setting, their observation that young adults more often receive multiagent chemotherapy regimens is concerning—these regimens have significant risk of toxicity, with >10% of patients experiencing persistent peripheral neuropathy and roughly 3% experiencing long-lasting toxicities that affect their ability to engage in activities of daily living.^17,25,26^ For these young patients with substantial life remaining, such complications may interfere with their ability to continue engaging meaningfully in their work, care for families/loved ones, and socialize with others, which may detrimentally affect quality of life.

To reduce rates of unnecessary chemotherapy use in stage II CC, efforts continue toward developing prognostic biomarkers that may help oncologists with risk stratification for individual patients. Circulating tumor DNA (ctDNA)–guided management may offer one potential remedy. Tie et al^27^ demonstrated the noninferiority of monitoring ctDNA levels after surgical resection to help determine the need for AC when compared with standard management, resulting in reduced rates of AC use with no impact on recurrence-free survival. Profiling microRNA (miRNA) expression is another promising avenue, with several studies demonstrating successful prediction of recurrence on the basis of miRNA expression patterns;^28,29^ in an ad hoc analysis from one of these works, patients in the high-risk miRNA group also experienced favorable response to chemotherapy.^29^ As these modalities grow more capable of differentiating patients at greatest risk of disease recurrence, it is our hope that oncologists will feel more confident using AC judiciously in stage II disease.

Several limitations of this study should be noted. Our greatest limitation is that all patients included eventually developed metastatic CC, potentially suggesting the presence of more aggressive tumors in this cohort. Although our findings do shed light on differential AC treatment practices in stage II disease that occurred without the knowledge that these patients would go on to develop metastatic disease, and while we highlight a lack of benefit for AC given during stage II on eventual outcomes in patients whose disease becomes metastatic, it should be noted that this characteristic may limit the generalizability of our findings. Data for high-risk features of CC including T4 disease, poorly differentiated histology, bowel obstruction or tumor perforation, fewer than 12 lymph nodes harvested at time of resection, and invasion of vascular, lymphatic, or perineural spaces were not available to determine the number of cases harboring high-risk features that may have justified adjuvant treatment in those who appear otherwise to have disease with lower risk of recurrence. Although TTMD is a reasonable substitute for progression-free survival (PFS), we did not have PFS or disease-free survival data available for inclusion in this work.

In conclusion, we showed bias in current treatment practices for stage II CC which favors providing postresection AC in younger patients over older patients, even in stage IIA disease, despite guidelines not routinely supporting this practice. A higher rate of treatment with AC in these patients who went on to develop metastatic disease was not found to be associated with better outcomes, including no difference in OS and a likely negligible difference in TTMD. These findings indicate that current treatment practices appear to be putting patients at higher risk of adverse events relating to chemotherapy treatment without providing any substantial benefit and may be disproportionately putting younger patients at higher risk. Clinicians must actively attempt to avoid such biases in care for patients with CC to avoid inducing preventable harm and unnecessary burden on these patients' lives relating to frequent clinic visits and the costs associated with more aggressive treatment that often is not warranted.

## APPENDIX

**TABLE A1.** Important Covariates Stratified by Age Group: Propensity Score Matched Data, All Stage II Patients

Covariate	Levels	LOCC	EOCC	*P*
Sex	Female	57 (54.3)	56 (53.3)	1.000
	Male	48 (45.7)	49 (46.7)	
Ethnicity	Hispanic or Latino	7 (6.7)	8 (7.6)	1.000
	Non-Hispanic	98 (93.3)	97 (92.4)	
Site	Colon	104 (99.0)	103 (98.1)	1.000
	Colorectal NOS	1 (1.0)	2 (1.9)	
ECOG	>1	3 (2.9)	3 (2.9)	.988
	0	72 (68.6)	71 (67.6)	
	1	30 (28.6)	31 (29.5)	

Abbreviations: ECOG, Eastern Cooperative Oncology Group; EOCC, early-onset colon cancer; LOCC, later-onset colon cancer; NOS, not otherwise specified.
